# Therapeutic Applications of Poly-miRNAs and miRNA Sponges

**DOI:** 10.3390/ijms26104535

**Published:** 2025-05-09

**Authors:** Cynthia Avendaño-Portugal, Mariela Montaño-Samaniego, Raquel Guttman-Bazbaz, Diana M. Bravo-Estupiñan, Miguel Ibáñez-Hernández

**Affiliations:** 1 Laboratorio de Terapia Génica, Departamento de Bioquímica, Escuela Nacional de Ciencias Biológicas, Instituto Politécnico Nacional, Carpio and Plan de Ayala, Col. Sto Tomás, Miguel Hidalgo, Mexico City 11340, Mexico; cynthia_aportugal@hotmail.com (C.A.-P.); mariela.mont3091@gmail.com (M.M.-S.); diana.marcela.bravo94@gmail.com (D.M.B.-E.); 2 Laboratorio de Técnicas Fototérmicas, Departamento de Ciencias Básicas, Unidad Politécnica Interdisciplinaria de Biotecnología, Instituto Politécnico Nacional, Mexico City 07340, Mexico; 3 Facultad de Ciencias de la Salud, Universidad Anáhuac México, Av. Lomas Anáhuac 46, Col. Lomas Anáhuac, Huixquilucan 52786, State of Mexico, Mexico; rakgutt@gmail.com; 4 Laboratorio de Quimiosensibilidad Tumoral, Facultad de Microbiología, Universidad de Costa Rica, San Jose 11501-2060, Costa Rica

**Keywords:** miRNA, RNA-based therapy, biomedicine, miRNA sponge, poly-miRNA

## Abstract

MicroRNAs (miRNAs) are small, non-coding RNA molecules that play crucial roles in regulating gene expression, and their dysregulation is implicated in various human diseases. Over the years, several research groups have identified miRNAs as promising therapeutic targets for intervention. Therapeutic strategies involve either overexpression or knockdown of specific miRNAs. This review aims to provide a comprehensive overview of synthetic poly-miRNAs and miRNA sponges, highlighting their therapeutic applications. It begins with an introduction to miRNAs and their role in human diseases, followed by a detailed discussion on synthetic poly-miRNAs and miRNA sponges by exploring their application in cardiovascular, inflammatory, autoimmune, and metabolic disorders, as well as in cancer therapy. Additionally, strategies for targeted delivery, challenges, and limitations of these therapies are addressed.

## 1. Introduction

MicroRNAs (miRNAs) are endogenous, small, conserved, and single-stranded non-coding RNA molecules of 21–25 nucleotides in length that play a crucial role in the post-transcriptional regulation of gene expression in eukaryotes. miRNAs bind to complementary sequences on their target messenger RNAs (mRNAs), which can lead to the degradation or translation inhibition of these mRNAs, thus regulating almost every important physiological process, such as development, embryogenesis, proliferation, apoptosis, cell growth, viral defense, differentiation, and metabolism [1,2,3,4].

The biogenesis of microRNAs (miRNAs) begins in the nucleus, where miRNA genes are transcribed by RNA polymerase II or III as long primary transcripts (pri-miRNAs). These transcripts fold into characteristic hairpin-like structures that are cleaved by the microprocessor complex, composed of Drosha and DGCR8, to produce ~70 nucleotide precursor miRNA (pre-miRNAs). miRNAs can be encoded in the genome either as individual transcriptional units—each giving rise to a single mature miRNA—or as miRNA clusters, where multiple miRNAs, sometimes with distinct sequences, are co-transcribed as a single pri-miRNA. These clustered miRNAs are typically processed into multiple mature miRNAs, which may act together or regulate different targets. Once processed in the nucleus, pre-miRNAs are exported to the cytoplasm via Exportin-5 (XPO5), where Dicer performs a second cleavage to generate a ~22-nucleotide miRNA duplex. One strand of the duplex is then incorporated into the RNA-induced silencing complex (RISC). Once loaded, the RISC uses the mature miRNA strand to recognize target mRNAs through the “seed region” (typically nucleotides 2–8 of the miRNA) and, in some cases, additional supplemental pairing regions. The Argonaute (AGO) protein, a core component of RISC, contains recognition chambers that facilitate target scanning and pairing. Upon stable miRNA–mRNA interaction, TNRC6 is recruited to the complex, leading to the recruitment of deadenylation complexes and subsequent mRNA degradation. Perfect complementarity between the miRNA and its target typically results in mRNA cleavage, while partial complementarity leads to translational repression and decay through deadenylation [5,6]. This pathway is tightly regulated and context-dependent, with evidence of tissue-specific, or even conditionally activated miRNAs [7].

Polycistronic miRNAs (poly-miRNAs) are engineered constructs containing multiple miRNA sequences processed from a single pri-miRNA, designed to mimic or improve upon the cooperative function of naturally occurring miRNA clusters [8,9,10]. While endogenous miRNA clusters are groups of miRNA genes located together in the genome and co-transcribed under the control of a shared promoter, synthetic poly-miRNAs can be designed to combine unrelated miRNAs, allowing for the tailored regulation of multiple pathways simultaneously [11,12,13]. This approach holds promise for multiplexed therapeutic strategies.

On the other hand, microRNA sponges (miRNA sponges) are a technology designed to bind and sequester miRNAs. These “sponges” contain multiple binding sites that are complementary to specific miRNAs (homo-sponges) or a group of miRNAs (hetero-sponges), acting as competitors for the binding of miRNAs and, therefore, inhibiting their function [14,15,16]. This technology allows the loss of miRNA-mediated repression of target mRNAs, which can be crucial in cases where miRNAs are overexpressed and contribute to the development and progression of disease. For instance, miRNAs have been associated with various pathological conditions, making them attractive targets for the treatment of cardiovascular, gastrointestinal, metabolic, neurodegenerative, and rheumatic diseases, as well as cancer and more. The ongoing development in this field is promising, with continuous improvements in the vehicles used for the delivery of poly-miRNAs and miRNA sponges, as well as the precision of targeting. In addition to their association with diseases, miRNAs have also been shown to play a role in the remodeling of the tumor microenvironment through their release in exosomes, mediated by cell-to-cell contact [17].

According to the NCBI database, in the past year, over 460,000 papers on miRNAs and their relation with multiple diseases have been published [18]. This growing body of evidence underscores the critical importance of investigating therapies targeting poly-miRNAs and miRNA sponges as a potential strategy for disease management and treatment. Hence, understanding the cooperative functions of miRNAs can offer a more comprehensive view of biological processes and diseases, leading to the identification of more precise therapeutic targets.

## 2. Overview of Synthetic Poly-miRNAs

**Figure 1:**
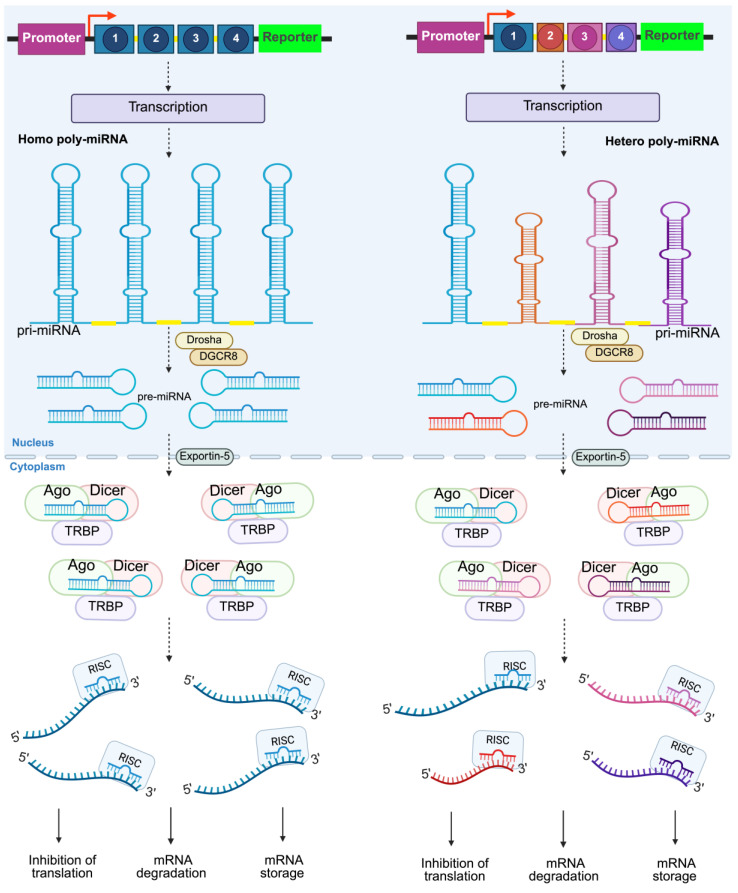
Schematic representation of poly-miRNA genetic constructs and their mode of action. The figure illustrates how poly-miRNA genes can be arranged within a genetic vector, driven by either a polymerase II or III promoter. On the **left**, an example of a homo poly-miRNA is shown, where identical miRNA sequences are repeated. On the **right**, a hetero poly-miRNA is depicted, where different miRNA sequences (represented in various colors) are arranged within the construct. After transcription, the poly-miRNA adopts a typical pri-miRNA stem-loop structure, with each stem-loop separated by a ‘spacer sequence’ (yellow). The pri-miRNA is then processed into mature miRNAs, which assemble with the RISC and bind to the 3′ UTR of target mRNAs, leading to mRNA decay. Created in BioRender. Portugal, (2025) https://BioRender.com/rnbsn8z.

As mentioned in the introduction, synthetic polycistronic miRNAs (poly-miRNAs) are inspired by naturally occurring miRNA clusters, which are transcribed as single units and processed into multiple functional miRNAs. These constructs may include multiple copies of identical miRNAs, forming a homo poly-miRNA—or different miRNAs —constituting a hetero poly-miRNA (Figure 1) [13,19]. Based on this principle, poly-miRNAs have been proposed as a therapeutic strategy to restore or enhance the expression of downregulated miRNAs associated with key cellular functions or disease-related phenotypes. This strategy can enhance their effects by amplifying the expression of a single miRNA or, in a synergistic manner, targeting multiple genes simultaneously, thereby making it feasible to modulate multiple genes by engineering a single transcript [10,13,19,20].

Poly-miRNAs are not the only strategy developed to restore miRNA function. Therapeutic approaches such as miRNA mimics and small interfering RNAs (siRNAs) are also widely explored to compensate for the loss of specific miRNAs or to silence pathological genes [13,21]. Additionally, short hairpin RNAs (shRNAs), which mimic the structure of pre-miRNAs, have been employed to achieve long-term knockdown effects. However, synthetic miRNA-based designs—such as poly-miRNAs—have been reported to offer certain advantages under specific conditions, including lower toxicity, stable and tunable expression, and the possibility of co-expressing multiple miRNAs or a gene of interest from a single transcript [13,21].

To achieve a potentially therapeutic poly-miRNA candidate, two main aspects should be considered: identifying the potential targets and constructing the backbone. Single miRNA prospect targets should be defined either by searching for known and fully characterized miRNAs in the miRBase and miRTarBase repositories and the literature or by identifying new targets through miRNA overexpression assays [22,23].

The efficiency of poly-miRNA backbones largely depends on recapitulating the essential elements required for proper miRNA biogenesis. The primary transcript (pri-miRNA) must contain structural and sequence features that facilitate precise and efficient processing by the microprocessor complex, composed of DROSHA, DGCR8, and additional cofactors [13,24]. Studies have identified specific sequence elements that enhance microprocessor recognition and cleavage, including a UG motif upstream and a CNNC motif downstream of the hairpin structure, which assist binding of cofactors such as SRSF3. Additionally, the GHG motif located in the lower stem is crucial for nucleotide-precise cleavage and serves as a structural signal for DROSHA binding. The overall structural integrity, including appropriate stem length and loop configuration, also contributes to processing efficiency. Computational analyses have further suggested that low Shannon entropy correlates with increased cleavage accuracy [6,25].

To enhance the expression and maturation of suboptimal miRNAs, the concept of cluster assistance has been described, wherein neighboring hairpins within the same transcript facilitate each other’s processing. Proteins such as the enhancer of rudimentary homolog (ERH) have been identified to support this cooperative maturation by interacting with helper hairpins [6].

Backbones from well-characterized natural clusters—such as miR-17-92 and miR-155—have been successfully employed in the construction of synthetic poly-miRNAs. For instance, the miR-17-92 backbone has been used as a scaffold to embed multiple synthetic miRNAs while maintaining efficient processing. This cluster is notable for containing the highest number of hairpins (six) within the shortest DNA segment among other native miRNA cluster loci. The complete process and steps for designing a poly-miRNA based on this backbone are described in detail by Bhaskaran et al. (2019); this approach leverages the endogenous architecture of polycistronic transcripts to improve stability and processing efficiency [24]. Similarly, the pri-miR-155 backbone has demonstrated high fidelity and robustness, enabling the generation of up to 18 functional miRNAs with diverse stem sequences within a single construct [13]. Additional design criteria include ensuring thermodynamic symmetry of the hairpin to favor loading of the antisense strand to the RISC, as well as sequence preferences such as A/U-rich 5′ ends and relatively low GC content [6,13,24].

To date, there have been at least two clinical trials for therapeutic microRNAs—miR-34 in gastrointestinal cancers [26] and miR-16 in mesothelioma [27]. Although never approved or even having entered phase III due to different concerns, the data suggested that the clinical impact may have been limited by the use of a single miRNA modulation, which may fail to recapitulate the complexity of a multigenic disease network [20]. In this regard, the work of Bhaskaran et al. confirms that microRNA clustering is a significantly more effective alternative due to a synergistic effect [20]. This approach has been applied to overexpress several inhibitory RNAs against cancer [28], hepatitis [29], influenza [30], and HIV [31]. Furthermore, it has been proved that it is achievable to transcribe multiple miRNA hairpins as a single transcript from the same Pol II promoter in mammalian cells (Figure 1). This confers an advantage since Pol II promoters are often stronger than others [13].

Poly-miRNAs provide a particularly appealing approach towards complex prophylaxis diseases, including cancer and other multifactorial disorders, as their pathogenesis is mainly driven by multigenic dysregulation [23,32,33]. By enabling the simultaneous regulation of several molecular targets, poly-miRNAs may offer a more comprehensive and potentially more effective therapeutic strategy [13,19,20,24].

It is worth noting that, although poly-miRNAs can function in a coordinated manner, evidence suggests that their main advantage lies in expanding the range of target genes, rather than intensifying the repression of shared targets [24].

## 3. Overview of Synthetic miRNA Sponges

Naturally occurring non-coding RNAs (ncRNAs) function as regulators of endogenous miRNAs known as competing endogenous RNAs (ceRNAs). The most studied ceRNAs are long non-coding RNAs (lncRNAs) and circular RNAs (circRNAs), both of which act as natural miRNA sponges. However, if the sequence of a specific mature miRNA targeted for downregulation is known, a synthetic miRNA sponge can be designed [34].

Like lncRNAs, miRNA sponges act as competitive inhibitors, sequestering miRNAs away from their target mRNAs. This competitive inhibition leads to loss of repression of mRNA targets, thereby altering protein synthesis and cellular function [34,35].

**Figure 2:**
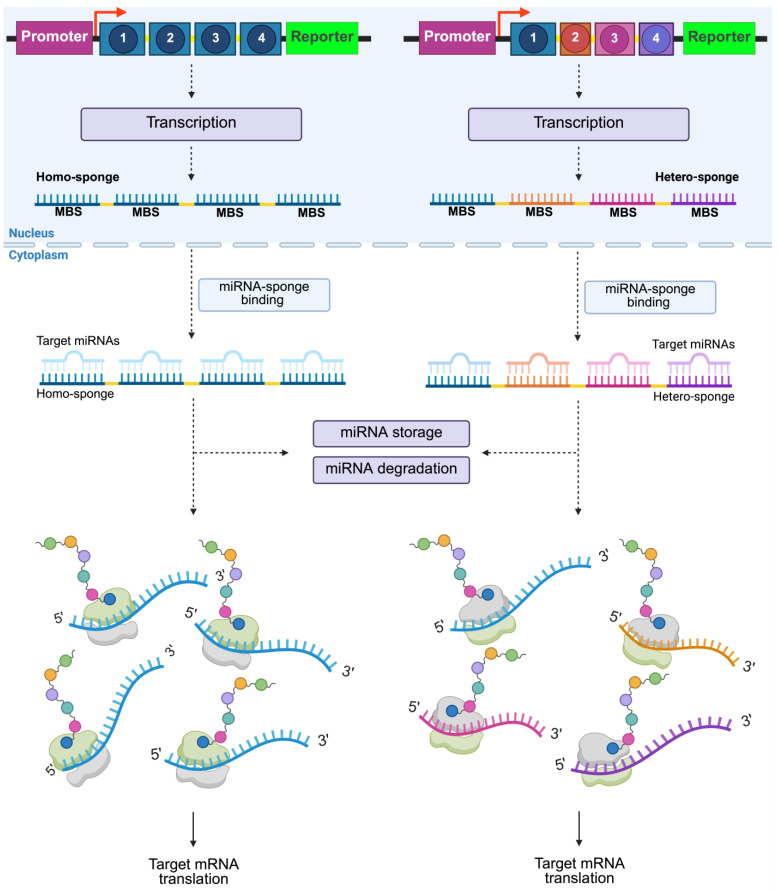
Schematic representation of synthetic miRNA sponges’ genetic constructs and their mechanism of action. The figure illustrates how miRNA sponge genes can be arranged within a genetic vector. On the **left**, an example of a homo sponge is shown, where identical MBSs are repeated. On the **right**, a hetero sponge is shown, where different MBSs (represented in various colors) are arranged within the construct; in both cases, the MBSs are separated by a spacer sequence (yellow). Once transcribed, the miRNA–sponge complex forms through complementary binding to the seed sequence of the miRNA, leading to the expression of target mRNA. Created in BioRender. Portugal, (2025) https://BioRender.com/79wvrsy.

A hallmark feature of miRNA sponges is the presence of multiple miRNA binding sites (MBSs). The formation of the miRNA–miRNA sponge complex sequesters the miRNA, preventing it from binding to its endogenous mRNA targets. This sequestration effectively diminishes the miRNA’s regulatory impact on gene expression, thereby relieving repression of downstream genes (Figure 2) [14,36,37].

The design of synthetic miRNA sponges is highly customizable. They can include MBSs that are either perfectly complementary to the mature miRNA of interest or contain bulged sections at the central position. It has been reported that perfectly base-paired miRNA sponges are vulnerable to cleavage by RNA-induced silencing complex (RISC) proteins, leading to rapid sponge degradation. In contrast, MBSs containing a 4-nucleotide bulge tend to be more stable [38,39]. Furthermore, non-perfect pairing sponges have better inhibition efficacy [35]. The MBSs can range from two to as many as the backbone and the structural properties of the transcript allow. Typically, they contain four to ten MBSs, as increasing the number may lead to diminishing returns [40]. On the other hand, a research group managed to successfully construct sponges with 16 binding sites for one specific miRNA and others with 32 binding sites targeting two different miRNAs. In expression experiments, the sponges containing 16 binding sites for two different miRNAs (32 in total) showed enhanced miRNA downregulation [35]. The effectiveness of these designs depends on the level of upregulation of the miRNA in a given tissue or disease; also, if more miRNAs share the same seed sequence, the biological effects may be amplified.

MBSs are arranged in a repetitive sequence pattern and separated by a “spacer sequence” typically consisting of four to eight nucleotides. This characteristic is customizable depending on the desired structure [35,38]. Additionally, one of the greatest qualities of synthetic miRNA sponges is that they can be designed to inhibit entire miRNA clusters by incorporating multiple distinct MBSs within a single construct (Figure 2) [38,40].

Synthetic miRNA sponges are transcribed from strong promoters in DNA vectors in an mRNA-like manner when transcribed by RNA polymerase II, though polymerase III promoters can also be used [41]. Polymerase II promoters offer advantages, such as the capping and polyadenylation of the resulting transcript. Also, the sponge sequence can be placed in the 3′ UTR of a reporter gene like EGFP. An additional advantage of polymerase II promoters for this purpose is that strong promoters like CMV, PGK, and EF1⍺ can be used, thus augmenting the expression of the transcript [38,39,40,42].

A key advantage of miRNA sponges over antisense oligonucleotides or antimiRs is their ability to interact with all members of an miRNA seed family. Since sponge–miRNA interaction relies on base-pairing within the seed region, the sponges can interact with all members of the miRNA seed family, whereas antisense oligonucleotides can only target one specific miRNA. Furthermore, many cell types, both in vitro and in vivo, exhibit resistance to oligonucleotide uptake. In contrast, for cell lines that are difficult to transfect or for in vivo applications, the sponge transgene can be introduced via a viral vector. By incorporating an open reading frame for a selectable marker or reporter gene within the vector, the selection, quantification, screening, fluorescence-activated cell sorting, or even laser capture microdissection of cells that strongly express the sponge can be facilitated. This enables the isolation of cells in which the miRNA family is significantly inhibited, allowing the detection of modifications in target gene expression [40].

Sponge stability and cellular localization can be optimized using chemical modifications and vector-based delivery systems [14,43,44]. Recent advancements in miRNA sponge technology have led to the development of inducible and tissue-specific sponges, which offer controlled and localized miRNA inhibition. These innovations minimize off-target effects and improve the therapeutic potential of miRNA sponges. Moreover, combination strategies integrating synthetic miRNAs and miRNA sponges have emerged, enabling the synergistic regulation of miRNA networks to achieve precise therapeutic outcomes [40].

Given their versatility, miRNA sponges have been widely explored for therapeutic applications in different diseases, particularly in conditions where specific miRNAs play a central role in disease progression. For instance, miR-21 sponges have been developed to counteract the overexpression of miR-21 in various cancers [45]. Similarly, sponges targeting miR-155 have shown potential in treating hematological malignancies, as miR-155 is implicated in the progression of leukemia and lymphoma [46].

In neurodegenerative diseases, miRNA sponges targeting miR-29 and miR-146a have been explored for Alzheimer’s disease therapy. These miRNAs are known to regulate pathways involved in neuroinflammation and amyloid plaque formation. By inhibiting these miRNAs, synthetic sponges can potentially alleviate disease progression and neuronal damage [47].

In cardiovascular research, miR-92a sponges have been investigated for their role in promoting vascular repair and angiogenesis. Since miR-92a negatively regulates endothelial function, its inhibition via sponges can enhance post-injury tissue regeneration and improve recovery outcomes [48].

As research progresses, synthetic miRNA sponges continue to show promise as versatile tools for dissecting miRNA functions and developing novel miRNA-based therapies. Their ability to modulate miRNA activity with high specificity underscores their potential in personalized medicine and targeted gene regulation strategies. The integration of miRNA sponges with emerging gene editing and RNA delivery technologies may further expand their clinical applicability, paving the way for next-generation therapeutics.

## 4. Applications in Cardiovascular Diseases

Cardiovascular diseases (CVD) are the most common cause of death worldwide; their prevalence continues to increase despite significant advances in primary prevention and therapeutic strategies over recent years [49,50]. Atherosclerosis is the most important risk factor for CVD and a complex disease that may lead to ischemic heart disease, hypertension, aortic aneurysm, and stroke [16,51]. Therefore, it is essential to thoroughly investigate cardiovascular disease’s molecular pathophysiology to discover new biomarkers that can improve early diagnosis and more efficient treatment for these diseases [2,3,52].

Several studies have reported the potential role of miRNAs in the cardiovascular system, such as in cardiac cell contractility and growth, angiogenesis, regulation of lipid metabolism, and plaque formation [53,54,55]. miRNA dysregulation has been observed in myocardial infarction [56,57], heart failure [58,59], and cardiac hypertrophy [49,60]; in both humans and animals, miRNAs are implicated in the development of atherosclerosis [3,61].

Numerous studies have examined the role of various miRNAs not only for diagnostic and prognostic purposes but also for treating several CVDs. For example, miR-33a/b is a proatherogenic miRNA that plays an important role in lipid metabolism and cholesterol homeostasis, impacting the progression of atherosclerosis [16,62]. Inhibiting the function of miR-33 is known to raise HDL levels and reduce atherogenic risk; however, long-term silencing and genetic knockout (KO) studies have shown that loss of miR-33 can lead to hepatic lipid accumulation, obesity, and metabolic dysfunction, particularly when combined with a high-fat diet (HFD). This is associated with increased food intake and impaired metabolic function in white adipose tissue and the liver [63,64]. Therefore, the effect of decreasing miR-33 in specific tissues or organs has been studied either as basic research or as a therapeutic proposal using miRNA sponges to preserve its antiatherogenic function, but omitting the well-known adverse effects [16,65], also offering potential therapeutic strategies for dyslipidemia [66].

Similarly, miR-122 has an important function in lipid metabolism and atherosclerosis development; however, it is also known to be elevated in heart failure patients [49,67,68]. Consequently, the use of miR-122 antagonists has been investigated. It was reported that the loss of miR-122 has anti-inflammatory effects in CVD and inhibits apoptosis of myocardial cells. In addition, miR-122 inhibition exerts antioxidant and anti-apoptotic effects on sepsis-induced myocardial injury [67,69]. It is important to note that long-term depletion of miR-122 is associated with steatohepatitis, fibrosis, and hepatocarcinogenesis, suggesting that complete depletion of miR-122 may have deleterious consequences in humans [70,71,72]. Therefore, additional research on heart-targeted inhibition is required.

miR-21 is one of the most studied miRNAs in CVD, given that it is the most prominent non-coding RNA associated with hypertension and atherosclerotic disease. miR-21 was upregulated in human atherosclerotic plaque and various heart diseases [73,74,75]. Consequently, inhibition of this miRNA—mainly by modified antisense oligonucleotides—has been evaluated and proven to be effective against cardiac fibrosis and dysfunction [76,77,78,79,80].

Previously, it has been suggested that suppressing miR-214-3p could be a potential therapy for myocardial infarction, while other investigations provided evidence that miR-214-3p contributes to ferroptosis and its inhibition in vivo attenuates cardiac ferroptosis and malfunction induced by myocardial infarction [57,81]. In the same way, an adeno-associated virus serotype 9 (AAV9)-mediated delivery of anti-miR-214 was proven to restore cardiac function in hypertrophic mouse hearts [82]. Although miRNA sponges have been developed for the suppression and even targeted delivery of specific miRNAs, alternative approaches that leverage naturally occurring ncRNAs are also emerging, such as the use of lncRNAs to inhibit miR-214 and restore the levels of its target genes, so it could be used to help alleviate osteoporosis [83,84]; however, there have been no studies on miRNA sponges for CVD treatment.

Another miRNA of special interest is miR-34a, whose expression is elevated in mouse hearts with myocardial infarction and aging, as well as in human failing hearts. Inhibition of miR-34 has protective properties in mice after cardiac insult [85,86,87,88]. miR-34 has been successfully regulated in the heart using an adenoviral approach [89], and considering this, it was possible to generate an miR-34 sponge for the heart. Nevertheless, the sponge activity did not achieve significant inhibition of miR-34a using targeted approaches compared to previous studies employing locked nucleic acid inhibitors [1,85]. Moreover, chronic inhibition of miR-34 may not be optimal since it has been reported to induce tumorigenesis [90]. Therefore, the development of a more efficient therapeutic approach directly targeting the heart to inhibit miR-34 is necessary.

miR-155 is upregulated in patients or animal models with cardiac inflammation and atherosclerotic lesions. The levels of miR-155 in the blood of people who died due to heart conditions were about four times higher than those who did not have heart problems [91,92,93]. It is known that miR-155 inhibition ameliorates cardiac inflammation and that it attenuates atherosclerosis development and progression in mice [78,92,94]. It has been suggested that macrophage-specific miR-155 inhibition could be a feasible therapeutic strategy to decrease atherosclerosis-related inflammation [91]. Conversely, Chung and colleagues, based on miRNAs that are naturally expressed in clusters, designed a polycistronic expression vector for RNA interference based on BIC/miR-155, although, without any specific therapeutic purpose, demonstrated that this strategy can be used to express multiple miRNAs from a single transcription unit, in order to increase the inhibition of a target RNA [95].

The ability of miRNAs to regulate multiple pathways involved in disease progression positions them as attractive therapeutic targets. However, the translation to the clinic requires overcoming significant barriers in delivery, specificity, and safety. Continued research is crucial to developing the full potential of miRNA-based therapies in CVD.

## 5. Role in Metabolic Disorders

### 5.1. Role of miRNAs and Applications in Metabolic Disorders

Metabolic disorders, including obesity, diabetes, and dyslipidemia, are complex diseases influenced by genetic and environmental factors, becoming a global health concern due to their increasing prevalence and association with serious complications such as CVD and cancer [51,96]. Traditional therapeutic approaches have limitations, prompting the search for novel strategies. Recent advances in the field of miRNAs highlight their crucial role in regulating metabolic processes and their potential as therapeutic targets [97].

### 5.2. Role in Obesity

Obesity is characterized by excessive fat accumulation and is a major risk factor for metabolic disorders, including diabetes and CVD [96,98]. Obesity leads to alterations in miRNA expression, and several miRNAs have been closely associated as regulators of adipogenesis and lipid metabolism, while also being dysregulated in obesity [99,100,101,102].

For example, the role of miR-33 in obesity, as mentioned briefly above, is being investigated. Tissue-specific KO models have demonstrated that loss of miR-33 in the liver does not affect body weight but improves insulin sensitivity and glucose homeostasis, and reduces lipid accumulation in the liver, thereby protecting it from metabolic dysfunction-associated liver disease (MASLD) [61,103]. In another study, miR-33 was deleted in AgRP neurons, leading to metabolic dysregulation and obesity in mice [104]. miR-33b serum levels are potential biomarkers for obesity and hypercholesterolemia [98], as well as possible treatments, underscoring the importance and need for more targeted miRNA treatments. Another well-established microRNA is miR-143, since it facilitates the differentiation of adipocytes [101,105,106,107], and its downregulation in cultured human pre-adipocytes leads to the inhibition of adipocyte differentiation [108], whereas the KO in brown adipose tissue significantly enhances thermogenesis, while in white adipose tissue inhibits the process of adipogenesis [109]. In obese mice, circRNF111 functions as a sponge for miR-143, suggesting a protective role of circRNF111 in metabolic syndrome, which may serve as a promising therapeutic target for mitigating lipid accumulation [110].

On the other hand, overexpression of miR-21 in white adipose tissue has been linked to obesity, as it regulates adipogenic differentiation [111,112]. In 2021, researchers found that administering an miR-21 mimic to obese mice prevented weight gain induced by a high-fat diet [111], confirming the potential of the miR-21 mimic as a therapeutic option for obesity. However, the outcomes remain unclear since it has been previously demonstrated that long-term inhibition of miR-21 reduced body weight and adipocyte size, while also improving lipid homeostasis [113]. Recently, an miR-21 sponge construct released by exosomes was developed, which has been shown to have the potential to suppress miR-21 and upregulate miR-21 target genes [114].

Elevated levels of miR-126 were found in obese children [115], aligning with studies showing that miR-126b overexpression promotes lipid deposition and exacerbates obesity symptoms in mice, leading to higher adipose tissue weight and insulin resistance [116,117]. Targeting miR-126 with a sponge-based approach could offer a potential therapeutic strategy for pediatric obesity.

### 5.3. Role in Diabetes and Insulin Resistance

Insulin resistance (IR) is a precursor defect in the vast majority of patients with type 2 diabetes mellitus (T2DM). It is characterized by dysfunctional insulin action, leading to increased insulin levels to maintain blood glucose levels within a normal range. This condition can result in negative outcomes such as obesity, hypertension, and dyslipidemia among patients with T2DM [112,118].

miR-21 appears to be involved in all metabolic disorders since its up-regulation is also associated with an increase in insulin resistance [119], and its downregulation via knockdown plasmids or sponges has been reported to be an effective therapy for diabetic nephropathy [120,121]. In addition, a research group indicated that an miR-21 antagomir was able to lower blood glucose levels and improve insulin resistance in T2DM rats [118]. More recently, a study revealed an increased level of miR-21 expression in diabetic patients and that the circRNA circ_0000652 acts as a sponge for miR-21; hence, it could be a novel therapeutic target for treatment, including the prevention of T2DM [122].

In contrast, reduced miR-146a levels have been linked with insulin resistance and reduced glycemic control, consistent with reports of miR-146a downregulation in obese and T2DM patients [112,123,124]. Several investigations have shown that miR-146a can mitigate the progression of diabetes complications, including nephropathy, wound healing [125], neuropathy, and retinopathy. Furthermore, injection of the miR-146a mimic has been confirmed to alleviate diabetes mellitus in animal models [123,126].

Other groups identified miR-690 as a key insulin-sensitizing miRNA that is highly expressed within exosomal-derived M2-polarized macrophages (M2 Exos). M2 Exos can improve insulin sensitivity in vivo [127,128]. Hence, if these results can be translated to humans, the miRNA contents of exosomes could potentially provide therapeutic options for treatment.

Another example of miRNA downregulation/silencing for insulin-resistant research is the silencing of miR-222, which reduces insulin-stimulated glucose uptake by ≈40% [129]. Although several studies suggest that miR-222 sponges can effectively inhibit miR-222 activity, these have been tested for other diseases rather than diabetes [130,131].

miR-223 is associated with the regulation of glucose homeostasis and inflammatory response. Two independent groups demonstrated that miR-223 is overexpressed in adipose tissue from women with IR or obese subjects [132,133], and that the overexpression of miR-223 in human adipocytes inhibits glucose uptake stimulated by insulin, leading to impaired insulin signaling and GLUT4 trafficking [132,134]. Although miR-233 sponges have been evaluated, and a sponge-mediated reduction level was achieved, these have not been specifically developed to evaluate their therapeutic potential in diabetes or IR [135,136]. In the same way, natural sponges such as lncRNAs and circRNAs can influence processes like kidney aging, cardiac hypertrophy, cancer progression, and liver fibrosis by modulating miR-223 activity [13,137,138,139,140], making them a promising strategy for the treatment of diabetes and IR.

### 5.4. Role in Dyslipidemia

Dyslipidemia, characterized by elevated lipid levels in the blood, is a common feature of metabolic disorders, and can lead to the development of obesity-associated diseases. Interestingly, IR is the primary catalyst for the development of dyslipidemia. miR-122, miR-27b, and miR-33 are involved in cholesterol and lipid metabolism but are dysregulated in dyslipidemia. Since the inhibition of miR-122 has been shown to reduce plasma cholesterol and triglyceride levels [141,142], many circRNAs—such as circ_0005963, circRNA_002581, circ_0007142, circ_0011269, and circ_0006404—have been proven to serve as a sponge of miR-122 [72,139,143,144]; hence, these miR-122 antagonists could be a promising strategy for the treatment of dyslipidemia, in addition to those mentioned in the previous section, with respective precautions due to concerns regarding the inhibition of miR-122 expression, as mice with either entire body or liver-specific deficiencies develop steatohepatitis, fibrosis, and hepatocellular carcinoma [145].

miR-27b, in contrast, acts as a regulator in lipid metabolism, influencing dyslipidemia by modulating the expression of key lipid-metabolism genes and affecting lipid accumulation, adipogenesis, and cholesterol metabolism [146,147,148]. In addition, it has been demonstrated that the inhibition of miR-27b was effective for reducing fat and body weight, decreasing levels of total cholesterol, triglycerides, and LDL-Ch, while increasing levels of HDL-Ch [148]. Recently, it has been reported that statin-induced miR-33a and miR-27b up-regulation contributes to insulin resistance and metabolic dysfunction, potentially leading to T2DM [149], reiterating the importance of miR-27b and its proper regulation in health and disease. Ongoing research is essential to developing the full potential of miRNA-based therapies in metabolic disorders, as they require overcoming significant barriers in specificity and safety.

## 6. Applications in Inflammatory and Autoimmune Diseases

Inflammatory and autoimmune diseases are characterized by dysregulated immune responses, often involving aberrant miRNA expression patterns. miRNA-based therapies, including synthetic miRNA sponges, hold great promise in modulating immune responses and restoring immune homeostasis.

Several studies have highlighted the role of specific miRNAs in the pathogenesis of inflammatory diseases. For instance, miR-146a and miR-155 are key regulators of immune-signaling pathways. While miR-146a functions as a negative regulator of inflammation by targeting TRAF6 and IRAK1, miR-155 promotes pro-inflammatory responses by modulating transcription factors such as NF-κB [150,151]. Synthetic miRNA sponges targeting miR-155 have been investigated as potential treatments for autoimmune conditions such as multiple sclerosis and rheumatoid arthritis, aiming to dampen excessive inflammation [152].

In systemic lupus erythematosus (SLE), miR-21 is upregulated and contributes to disease progression by enhancing T-cell activation and reducing immune tolerance. An miR-21 sponge-based approach has been proposed to mitigate this hyperactivation and restore immune balance [153]. Similarly, miR-223, which is implicated in inflammatory bowel disease (IBD), is a promising target for sponge-based interventions to regulate intestinal immune responses [154].

Beyond direct miRNA inhibition, tissue-specific and inducible miRNA sponges are being developed to minimize off-target effects and improve therapeutic precision. These advanced sponges are particularly valuable in chronic inflammatory diseases, where long-term regulation of immune pathways is necessary.

Various synthetic miRNA sponges have been designed for different diseases, demonstrating significant therapeutic potential. For example, miR-21 sponges have shown promise in systemic lupus erythematosus (SLE) by sequestering miR-21 and restoring immune function, leading to reduced autoantibody production and alleviation of lupus symptoms [155]. Similarly, miR-155 sponges have been utilized in multiple sclerosis and rheumatoid arthritis to reduce inflammatory cytokine production and mitigate disease progression [152].

In the context of neuroinflammatory disorders, where miR-326 is often downregulated, synthetic miR-326 sponges have been used to fine-tune immune responses, balancing pro- and anti-inflammatory pathways and improving survival rates [156]. Cardiovascular disease has also been targeted using miRNA sponges, with miR-92a sponges developed to enhance vascular repair and angiogenesis, showing positive outcomes in models of myocardial infarction and stroke [157].

Other applications include antiviral therapy, such as miR-122 sponges that have been investigated for blocking hepatitis C virus (HCV) replication, resulting in reduced viral load and improved antiviral responses [158].

Neurodegenerative diseases such as Alzheimer’s disease have been targeted with miR-29 sponges, which regulate pathways involved in neuroinflammation and amyloid plaque formation, leading to improvements in cognitive function [159]. Fibrotic diseases have seen potential therapeutic applications with miR-200 sponges, which reduce fibrosis progression in lung and liver fibrosis models [160].

Finally, in osteoarthritis, miR-16 sponges have been explored to regulate joint inflammation and cartilage degradation, offering a novel approach to improving joint health [161].

As miRNA sponge technologies continue to evolve, their integration with nanoparticle delivery systems and gene therapy approaches is expected to enhance their clinical viability. The potential to fine-tune immune responses through miRNA sponges positions them as an innovative and promising strategy in the treatment of inflammatory, autoimmune, and other chronic diseases.

## 7. Cancer Therapeutics

Despite significant progress in early detection and treatment strategies, cancer remains one of the leading causes of death worldwide—ranking second—and its incidence continues to rise. It is, therefore, imperative to continue searching for innovative treatments. In this context, it has been proven that miRNAs can repress hundreds of genes and regulate a wide range of cellular pathways, including those involved in cancer progression [162,163,164]. For instance, miRNAs can target the mRNA of tumor suppressor genes (oncomiRNAs) or oncogenes (tumor-suppressor miRNAs), thereby modulating processes such as metastasis, angiogenesis, epithelial–mesenchymal transition (EMT), migration, invasion, cell proliferation, immune escape, and chemoresistance [162,165]. By modulating the imbalance of miRNAs in cancer therapy, huge benefits can be achieved—from elucidating the heart of cancer pathogenesis to reducing metastasis and enhancing immune response [166,167,168]. Therefore, these therapies can function as an adjunct or even a replacement for conventional chemotherapy and radiotherapy [169,170].

For example, oncomiRNAs such as miR-21 have been reported to be upregulated in several cancer types, including melanoma, glioblastoma, colorectal, lung, stomach, prostate, pancreatic, and breast cancer. This upregulation promotes tumor growth, inhibition of cell death, and chemoresistance [171]. In 2015, an antimiR-21 sponge with three binding sites encoded in plasmid DNA was delivered to MCF-7 breast cancer cells. A lower expression of miR-21 was observed, which resulted in G1-phase cell cycle arrest, activation of the caspase-3 apoptosis pathway, and sensitization to doxorubicin and cisplatin [172]. Using a sponge targeting one oncomiRNA has proven to be a good strategy. However, based on these findings, researchers have explored sponges that simultaneously target multiple oncomiRNAs. For instance, Jie et al. constructed sponges with multiple binding sites for two miRNAs by developing mir-21/miR-31 and miR-31/miR-155 sponges with 16 binding sites for each miRNA [35]. miR-31 is notably overexpressed in lung adenocarcinoma, squamous cell carcinoma, and large cell lung carcinoma, and this overexpression is associated with primary tumor growth and metastasis, since it promotes activation of the RAS/MAPK pathway [173]. Conversely, miR-155 induces resistance to chemotherapeutics, and its downregulation in an orthotopic lung cancer model resensitizes tumors to chemotherapy. Also, miR-155 is linked to a downregulation of TP53, and its low expression is associated with shorter survival in lung cancer [174]. The sponges developed in that study proved effective in lung cancer cell lines by inhibiting cell growth, with their effects being more pronounced than those observed with conventional miRNA inhibitors. In addition to these examples, the miR-221/222 cluster plays a crucial role in cancer progression. Sharing the same seed sequence and targeting the mRNA of the cell cycle regulator p27^kip1^, the overexpression of miR-221/222 promotes cell proliferation in thyroid papillary carcinoma, breast cancer, lung cancer, and hepatocellular carcinoma (HCC) [175]. As an alternative therapy for HCC—the second leading cause of cancer death in men [176]—genetically modified adeno-associated viral vectors (AAV) were developed to express a sponge with four binding sites for miR-221. When tested in HCC cell lines, the sponge repressed the expression and activity of miR-221, leading to increased apoptosis [177]. Moreover, as p27^kip1^ mRNA is not the only target of the miR-221/222 cluster, these oncomiRNAs have also been implicated in tamoxifen resistance in breast cancer [178]. Considering this, another group designed an miR-221/222 sponge to inhibit their activity in tamoxifen-resistant MCF-7 cells, observing that the sponge represses the miR-221/222 expression and, consequently, abrogates tamoxifen resistance by restoring ER expression⍺ [179].

On the other hand, tumor-suppressor miRNAs are often downregulated in cancer, resulting in the overexpression of genes involved in tumor growth, cell survival, and antiapoptotic proteins [180]. Restoring the levels of the type of miRNAs has demonstrated positive biological effects in cancerous tissues. For instance, miR-124 has been identified as a tumor suppressor across cancer, including glioblastoma [181,182]. Glioblastoma is known to be the most aggressive primary malignant brain tumor, with a median survival of less than 2 years [183]. In 2023, several tumor-suppressor miRNAs were identified on clinically relevant glioblastoma cell lines and proposed as candidates for miRNA-based therapy. As a result, a poly-miRNA to overexpress miR-124-2, miR-135a-2, and let-7i was constructed. Consequently, the study found that using the poly-miRNA was more effective against glioma than individually delivered miRNAs. The enhanced efficacy can be attributed to the high heterogeneity of glioblastoma tumors, where more than one molecular subtype may coexist; therefore, determining and overexpressing pan-subtype miRNAs can represent a better chance for therapeutic success [10]. In this context, miR-135a-2 regulates cell proliferation and invasion via MAPK and JAK/STAT3 pathways and inhibits EMT [184], while let-7i inhibits UDP-Galactose-4-epimerase, which is overexpressed in glioblastoma cells, promoting cell proliferation and migration [185].

Furthermore, poly-miRNAs represent a promising approach for cancer treatment but can also be integrated as tools to enhance other advanced therapies. For example, they may improve the development and efficacy of CAR T cell therapy, where T cells are genetically modified to express a chimeric antigen receptor that recognizes tumor antigens, and its recognition triggers the cascade of activation for the T cell [17,186]. CAR T cell therapy has been proven effective but has disadvantages, such as the population of CAR T cells decreasing over time. It has been observed that CAR T cells used for successful treatments have shown an increase in mitochondrial mass and fusion events and a reduction in anaerobic metabolism, thus, promoting these events in CAR T cells using a cluster of miRNAs—such as poly-miRNAs—could improve the efficacy of CAR T cell therapy while offering a safe, non-antigenic alternative [17].

## 8. Targeted Delivery Approaches

Targeted delivery is still a major issue in gene therapy applications, as it is crucial to enhance specificity while minimizing the off-target effects [187]. Gene delivery strategies are classified into viral and non-viral vectors, both of which can be engineered to improve targeting efficiency.

Viral vectors, such as adeno-associated viruses (AAVs), lentiviruses, and retroviruses, exhibit superior delivery capacity compared to non-viral vectors [188]. However, achieving tissue-specific targeting requires advanced modifications. Strategies to enhance viral vector specificity include capsid engineering, ligand functionalization, and the use of cell-specific promoters. Capsid engineering modifies the aminoacidic sequence of the viral capsid to improve receptor affinity, while ligand functionalization involves attaching molecules such as antibodies, peptides, or aptamers to facilitate receptor-mediated endocytosis [189,190,191]. Nevertheless, the main challenge of using these vectors is biosafety in clinical translation, as their use may be highly immunogenic, which can impact the therapy’s safety, durability, and efficacy [188]. Another major challenge to overcome is genotoxicity associated with some viral vectors, such as gammaretroviral vectors, which integrate into the cell–host genome. This integration may be advantageous when stable, long-term expression is needed; however, it also comes with risks, as the inserted genes tend to integrate near transcriptional start sites and have an affinity for oncogenes [192].

On the other hand, non-viral vectors such as lipid nanoparticles (LNPs), liposomes, and exosomes offer an alternative with lower immunogenicity, cytotoxicity, and mutagenesis [193,194]. When delivering DNA or RNA, cationic lipids have proven to be the most effective [195]. Their targeting ability can be enhanced through surface modification, charge-based targeting, and microfluidic-based engineering. LNPs and liposomes can be conjugated with antibodies, aptamers, or peptides for site-specific delivery, while cationic lipids improve nucleic acid encapsulation and enhance cellular uptake, particularly for RNA-based therapies [196]. The disadvantages of non-viral vectors include high production costs and possible cytotoxic effects. Several research groups are still in the race to find more effective and secure strategies to overcome the first one, such as using microfluidic devices to produce nanovesicles for nucleic acid delivery. These devices create precise flow-controlled environments, making them ideal in terms of batch-to-batch reproducibility since they minimize the intermediate steps required in conventional method production [197].

Whether using viral or non-viral vectors, specificity can be achieved by using tissue-specific promoters, which are promoters that are only active in specific cell tissues. Since miRNA sponges and poly-miRs are coded in an mRNA-like manner, this approach may be more effective for targeted therapy since its use can restrict unwanted transgene expression [187,198]. Tissue-specific promoters have been used in several in vitro and in vivo studies. For example, the CC10 promoter restricts transgene expression to lung cells [199]; cytokeratin 18 and 19 promoters are specific to epithelial cells; the kallikrein promoter is specific for salivary glands [198]; and the PEPCK promoter is liver-specific [16], among others. One disadvantage of tissue-specific promoters is that many of them are relatively weak compared to viral promoters like CMV or SV40. However, several studies have developed methods to improve their activity without losing their specificity, such as creating chimeric promoters using parts of strong promoters, combining two specific promoters, or adding elements like TATA boxes [200,201].

Recent advances in ligand-conjugate delivery systems also offer promising tools to enhance cell-type specificity in miRNA-based therapies, particularly for non-viral approaches [202,203]. Although not the focus of this review, continued development in this area may complement promoter-based targeting strategies and broaden the translational potential of synthetic miRNA tools.

In summary, while the targeted and effective delivery of genetic material remains the most significant challenge in gene therapy applications, numerous research groups continue exploring improved techniques from different perspectives—optimizing genetic constructs, modifying delivery vehicles, or refining extraction techniques. The continued acquisition of knowledge in this area is crucial for advancing the clinical application of these therapies.

## 9. Challenges and Limitations

Despite promising preclinical results, miRNA therapeutics have faced a challenging path in the clinical field since no miRNA-based drugs have been approved or even in clinical phase III [23,32,33,204,205]. The first in-human trial of miRNA therapy was MRX34, an miR34a mimic launched in 2013. It was halted in 2016 due to severe toxicity and safety issues, and four patient deaths [23,26,32,206]. Since then, multiple miRNA drug candidates have entered early-phase clinical trials, including miR-16 mimic (TagomiRs) (28) [27], antimiR-155 (Cobomarsen) [207], antimiR-221 (MesomiR-1) [207], Pri-miR-451 (AMT-130) [208], and miR-193a-3p mimic (INT-1B3) [209], but none have advanced to phase III [205]. Many of these trials were terminated early due to insufficient efficacy, delivery issues, safety concerns, and insufficient funds. For example, the Cobamarsen trial was terminated in 2021 despite initial promise in hematological malignancies. These examples highlight how the translation of miRNA-based therapies into the clinic remains limited despite encouraging preclinical data [23,205].

Beyond clinical setbacks, designing poly-miRNAs and miRNA sponges with high on-target activity and minimal off-target effects remains challenging due to the complex regulation of miRNA biogenesis and the intrinsic pleiotropic nature of miRNAs. These small RNAs are capable of recognizing multiple targets, therefore triggering different molecular mechanisms and pathways depending on the site or context [20,32,210]. In cancer, several miRNAs have shown a potential dual effect as both oncomiRNAs and tumor-suppressors, depending on the tissue type, tumor microenvironment, or disease stage [206,211]. For example, miR-10b can act differently depending on cancer type [206], while the function of miR-200c depends on the carcinogenic stage [211]. Another widely studied example is miR-21, which promotes oncogenesis in various cancers by targeting tumor suppressors [212], yet plays a protective role in fibrotic diseases [213]. Although miRNA sponges and poly-miRNAs are designed to enhance specificity by targeting particular miRNAs or clusters, their regulatory effects can still be pleiotropic, yielding divergent outcomes across distinct cell types or cell cycle phases [42,205].

Toxicity and off-target effects remain major limitations. While poly-miRNAs offer the potential to modulate multiple genes simultaneously, they also increase the risk of off-target interactions and unintended biological effects. This is particularly critical in therapeutic contexts, where such interactions may lead to systemic toxicity of immunogenicity [21,26]. Moreover, the translation of these platforms into therapeutic applications is hindered by the difficulty in predicting all potential off-target interactions in vivo. A notable editorial from *Nature Biotechnology* (November 2024) highlighted that off-target effects remain the central bottleneck preventing miRNA drugs from reaching the market [23].

In 2021, Zhang et al. evaluated 10 miRNA drug prospects (none of which reached phase III) to assess their miRNA targets and compared them to those of siRNA drug prospects. They found that for every miRNA, the target number ranged from tens to hundreds. In contrast, siRNA targets oscillated only between 1 and 3, thus shedding light on some of the hurdles that miRNA-based therapies have faced to become marketable or even go further in clinical trials. On a different assessment, the same group points out that the target number of each FDA-approved drug (since 1939) is no more than five [214], providing perspective on where miRNA molecules stand in the clinical field and the broader RNA-based therapy race.

Moreover, the off-target particularity has also been related to neuro- and immunotoxicity [32,206,215]. To overcome these limitations, advanced tools such as single-cell RNA sequencing and AI-driven target prediction algorithms have been proposed as promising strategies. These approaches can enhance the identification of context-specific miRNA–mRNA interactions, allowing for more precise functional predictions and reduced off-target effects in therapeutic applications [216,217].

These concerns could be mitigated by targeting the effects of these constructed poly-miRNAs and miRNA sponges, whether through targeted delivery (including on-site administration, encapsulation in selective tropism-driven vectors and/or antibody-mediated targeting [27,218]), or through targeted expression by using tissue or tumor-specific promoters to modulate transcription in the desired site, even when systemically administered [187,219].

Nevertheless, the delivery of nucleic acids still represents a major obstacle for the development of therapeutic miRNAs, as it is imperative to overcome biological and pharmacological barriers—including stability in physiological environments, susceptibility to degradation, and the physical challenges of crossing biological membranes [211]. Therefore, effective delivery systems are crucial for translating miRNA-based therapies into clinical applications, whether using viral or non-viral vectors. In this regard, innovative package-free alternatives, such as ligand-conjugated miRNAs, are being explored [219]. Hence, for miRNA-based therapies to succeed in the clinic, delivery must be addressed so that miRNA molecules reach the desired cells, bypassing healthy cells and immune recognition [32].

Another major limitation is the accurate identification of functional targets. As the miRNA annotations on the MiRBase are often incomplete or inconsistent, this leads to poor concordance when comparing overexpression/inhibition experiments in vitro with the observations in vivo [23,220].

As outlined in Section 2 and Section 3, constructing a functional poly-miRNA or miRNA sponge sequence is difficult on its own. For poly-miRNAs, in silico analyses, folding predictions, and processing might result differently than anticipated [24]. In addition, it has been suggested that as the number of microRNAs encoded within the mRNA-like transcript increases, the expression of mature microRNA decreases, possibly due to a reduced cleavage efficacy in the microprocessor complex [24].

Moreover, when considering miRNA-based solutions for therapeutic purposes, it is important to note how the pathogenesis occurred in the first place. As stated before, many complex diseases develop due to the altered regulation of microRNAs, which, among several factors, could be influenced by alterations in the biogenesis, processing, and cleavage of miRNAs. Consequently, this prevents therapeutic miRNAs from providing effective solutions [32,206,221,222,223,224,225]. Although most of this is in cancer, the same might hold true in every other disease, as it is widely accepted that miRNA dysregulation plays a significant role in pathogenesis and disease progression [32,33,206,221].

In contrast to poly-miRNAs and miRNA sponges, emerging approaches such as CRISPR-based miRNA editing and antagomiRs offer alternative mechanisms to modulate miRNA activity with potentially higher precision. Antagomirs exhibit improved stability and tissue distribution, though they often require repeated administration [226]. Meanwhile, CRISPR-based strategies allow for direct editing of miRNA genes or regulatory regions, but face delivery and ethical challenges, particularly in somatic contexts [227]. Despite these differences, all miRNA-targeting approaches share common regulatory hurdles. Guidelines from the FDA and EMA for RNA-based therapies emphasize the need for rigorous assessment of off-target effects, immunogenicity, and long-term safety. The complexity of miRNA biology, combined with their pleiotropic functions, poses specific challenges for meeting these regulatory standards [228,229].

Therefore, it is imperative to continue the search for better bioinformatic tools and to conduct more research on specific miRNAs. This will enhance our understanding of the pathways in which they are involved and, in turn, enable the development of safer therapeutic strategies.

## Data Availability

No new data were created or analyzed in this study. Data sharing is not applicable to this article.

## Abbreviations

The following abbreviations are used in this manuscript:

RNA	Ribo nucleic acid
mRNA	Messenger RNA
miRNA	MicroRNA
Pol	Polymerase
Pri-miRNA	Primary micro-RNA
Pre-miRNA	Precursor micro-RNA
NCBI	National Center for Biotechnology Information
siRNA	Small interfering RNA
shRNA	Short hairpin RNA
ncRNA	Non-coding RNA
ceRNA	Competing endogenous RNA
lncRNAs	Long non-coding RNA
circRNAs	Circular RNA
AGO	Argonaute
XPO5	Exportin 5
RISC	RNA-induced silencing complex
TNRC6	Trinucleotide repeat-containing gene 6A protein
Poly-miR	Polycistronic miRNA
MBS	Multi-binding site
CVD	Cardiovascular disease
DGCR8	DiGeorge syndrome critical region 8 protein
SRSF	Serine/arginine-rich splicing factor
ERH	Enhancer of rudimentary homolog
KO	Knock out
HFD	High-fat diet
AAV	Adeno-associated virus
BIC	B-cell integration cluster
MASLD	Metabolic dysfunction-associated liver disease
AgRP	Agouti-related peptide
IR	Insulin-resistant
T2DM	Type 2 diabetes
GLUT4	Glucose transporter type 4
LDL-Ch	Low-density lipoprotein cholesterol
HDL-Ch	High-density lipoprotein cholesterol
NF-κβ	Nuclear factor kappa B
SLE	Systemic lupus erythematosus
IBD	Inflammatory bowel disease
HCV	Hepatitis C virus
EMT	Epithelial–mesenchymal transition
HCC	Hepatocellular carcinoma
LNP	Lipid nanoparticle
